# Does Glycosylation as a modifier of Original Antigenic Sin explain the case age distribution and unusual toxicity in pandemic novel H1N1 influenza?

**DOI:** 10.1186/1471-2334-10-5

**Published:** 2010-01-07

**Authors:** Tom Reichert, Gerardo Chowell, Hiroshi Nishiura, Ronald A Christensen, Jonathan A McCullers

**Affiliations:** 1 Entropy Research Institute, 345 S. Great Road, Lincoln, 01773, Massachusetts, USA; 2 Mathematical, Computational & Modeling Sciences Center, School of Human Evolution and Social Change, Arizona State University, Tempe, 85287, Arizona, USA; 3 Division of Epidemiology and Population Studies, Fogarty International Center, National Institutes of Health, 31 Center Dr-MSC 2220, Bethesda, 20892-2220, Maryland, USA; 4 PRESTO, Japan Science and Technology Agency, Saitama, 332-0012, Japan; 5 Theoretical Epidemiology, University of Utrecht, 3584 CL Utrecht, The Netherlands; 6 Department of Infectious Diseases, St. Jude Children's Research Hospital, 262 Danny Thomas Place, Memphis, 38104, Tennessee, USA

## Abstract

**Background:**

A pandemic novel H1N1 swine-origin influenza virus has emerged. Most recently the World Health Organization has announced that in a country-dependent fashion, up to 15% of cases may require hospitalization, often including respiratory support. It is now clear that healthy children and young adults are disproportionately affected, most unusually among those with severe respiratory disease without underlying conditions. One possible explanation for this case age distribution is the doctrine of Original Antigenic Sin, i.e., novel H1N1 may be antigenically similar to H1N1 viruses that circulated at an earlier time. Persons whose first exposure to influenza viruses was to such similar viruses would be relatively immune. However, this principle is not sufficient to explain the graded susceptibility between ages 20 and 60, the reduced susceptibility in children below age 10, and the unusual toxicity observed.

**Methods:**

We collected case data from 11 countries, about 60% of all cases reported through mid-July 2009. We compared sequence data for the hemagglutinin of novel H1N1 with sequences of H1N1 viruses from 1918 to the present. We searched for sequence differences that imply loss of antigenicity either directly through amino acid substitution or by the appearance of sites for potential glycosylation proximal to sites known to be antigenic in humans. We also considered T-cell epitopes.

**Results:**

In our composite, over 75% of confirmed cases of novel H1N1 occurred in persons ≤ 30 years old, with peak incidence in the age range 10-19 years. Less than 3% of cases occurred in persons over 65, with a gradation in incidence between ages 20 and 60 years.

The sequence data indicates that novel H1N1 is most similar to H1N1 viruses that circulated before 1943. Novel H1N1 lacks glycosylation sites on the globular head of hemagglutinin (HA1) near antigenic regions, a pattern shared with the 1918 pandemic strain and H1N1 viruses that circulated until the early 1940s. Later H1N1 viruses progressively added new glycosylation sites likely to shield antigenic epitopes, while T-cell epitopes were relatively unchanged.

**Conclusions:**

In this evolutionary context, Original Antigenic Sin exposure should produce an immune response increasingly mismatched to novel H1N1 in progressively younger persons. We suggest that it is this mismatch that produces both the gradation in susceptibility and the unusual toxicity. Several murine studies suggest specific cell types as a likely basis of the unusual toxicity. These studies also point to widely available pharmaceutical agents as plausible candidates for mitigating the toxic effects. The principle of Original Antigenic Sin modified by glycosylation appears to explain both the case age distribution and the unusual toxicity pattern of the novel H1N1 pandemic. In addition, it suggests pharmaceutical agents for immediate investigation for mitigation potential, and provides strategic guidance for the distribution of pandemic mitigation resources of all types.

## Background

The currently expanding worldwide spread of the swine-origin influenza novel H1N1 virus has produced infections with an unusual age distribution. Early reports suggest a high attack level for persons between the ages of 5 and 30 years of age with a peak at the age group 10-19 years, a decline in attack rate with age from age 30 to 60, and very low levels for persons over age 60.

While the virulence of novel H1N1 has been generally low thus far, comparable in most cases to that associated with recent seasonal influenza, two remarkable features have been seen:

1. A relatively large number of cases of young persons with severe respiratory disease.

2. About 35-45% of these have had no underlying medical conditions.

We suggest that this unusual age distribution and toxicity pattern can be explained by discernible features in the evolution of human H1N1 viruses and the timing of first exposure to such viruses. If the distribution of cases continues to follow the same pattern, our results should inform administrative policy, suggest specific non-pharmaceutical interventions to mitigate pandemic spread and direct clinical inquiry into specific mechanisms and possible therapeutic options.

## Methods

### Data Used

#### Age distribution data

We collected and summarized the latest data on the age distribution of confirmed cases of novel H1N1 and severe respiratory disease available as of July 2009.

[North America (Mexico [1] and Canada [2]), the European Union (EU/EFTA)[3], South America (Argentina [4], Chile [5], and Peru [6].), Oceania (Australia [7] and New Zealand [8]), and Asia (Japan [9] and Thailand [10])]. Early information on severe disease is from the World Health Organization [11]. The number of cases for which the age distribution has been reported for each country is included in a separate table (see Additional File 1). Data for the country with the largest number of cases, USA, are included in Additional File 1, but could not be included in the Figure because the age bins that appear in all US reports are neither uniform in age span nor as fine-grained as those used by all other countries. Nevertheless, US data are completely consistent with the composite developed here.

#### Virus sequence data

All human H1N1 viruses with publicly available, full-length sequences of the hemagglutinin subunit HA1 from 1918 to 1957, and a selection of representative sequences from every year thereafter including the 2009 novel H1N1 were studied (see Additional File 2 for list and accession numbers). Nucleotide sequences for entire hemagglutinin (HA) were obtained from the Influenza Research Database [12,13].

### Tools and Methodology

Predicted structures were generated and manipulated with *Jmol *software within the Influenza Research Database. Phylogenetic trees were constructed using the neighbor-joining method and bootstrap analysis (n = 500) to determine the best-fitting tree [14]. Nucleotide distances were estimated and evolutionary trees drawn as described [14]. Sites for potential glycosylation were determined by examination of the predicted amino acid sequences of HA for the N-X-S/T sequon (X = not proline).

## Results

**Figure 1:**
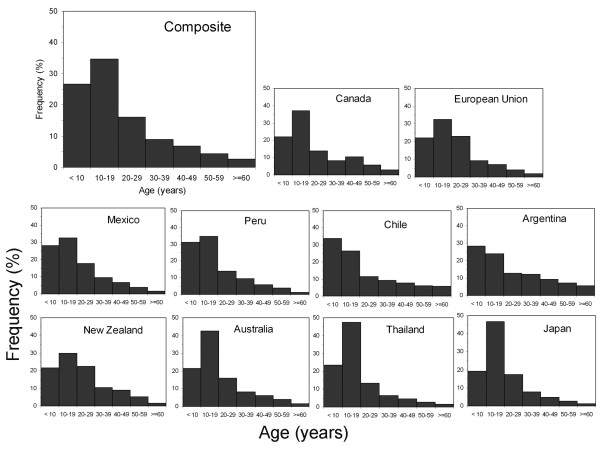
**The age distribution of frequency of cases of novel H1N1 in ten countries on five continents confirmed through the last date available in July, 2009**.

From the assembled data, we created a composite age distribution of confirmed cases of novel H1N1 through the latest date available (in most countries, mid- to late-July 2009) for 10 countries on five continents (Figure 1). For every dataset, we have yet to observe the entire course of this wave of the pandemic. Nevertheless, Figure 1 captures the early dynamics in each country and demonstrates that the initial case structure has been driven by the most vulnerable age-group, age 10-19. Arranged by geographical region, and within region by shape variation, Figure 1 demonstrates that country-to-country variability is not large; and what differences there are develop consistently within a region. Overall ~75% of the cases have been confirmed in persons below age 30 with a modest peak at the 10-19 age group. Fewer than 3% of the cases have occurred in the elderly ≥ age 65. The drop in incidence after age 20 is marked and uniform.

### Relation of novel H1N1 to other human viruses

**Figure 2:**
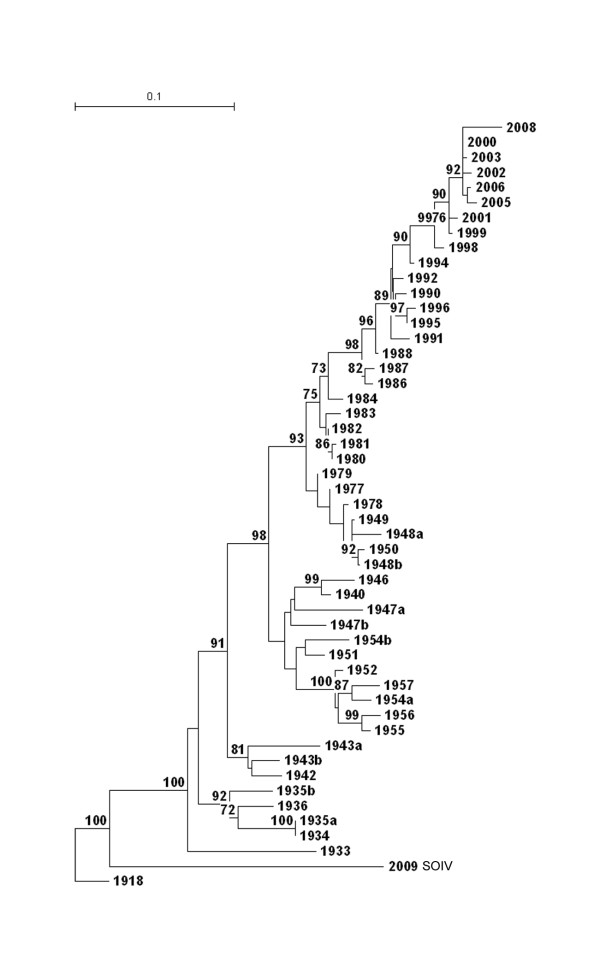
**Phylogenetic analysis of the HA1 of H1N1 influenza viruses**. Phylogenetic tree constructed using the neighbor-joining method and bootstrap analysis (n = 500) to determine the best-fitting tree for the HA1 region of the HA gene. Selected, relevant strains of the H1N1 subtype are listed by year of isolation. Small case "a" and "b" designate when two distinct strains co-circulated in the same year. Bootstrap values ≥ 70% are shown at branchpoints.

A phylogenetic tree based on the nucleotide sequence of representative human influenza viruses that circulated between 1918 and 2009 was generated (Figure 2). Among human viruses, HA of novel H1N1 is most closely related to the 1918 pandemic strain and descendants of this virus that circulated in the 1930s through 1943. Viruses that circulated after the re-emergence of H1N1 in 1977 are most closely related, by HA sequence, to strains that circulated between 1948 and 1950.

### Evolution of H1N1 HA glycosylation

**Figure 3:**
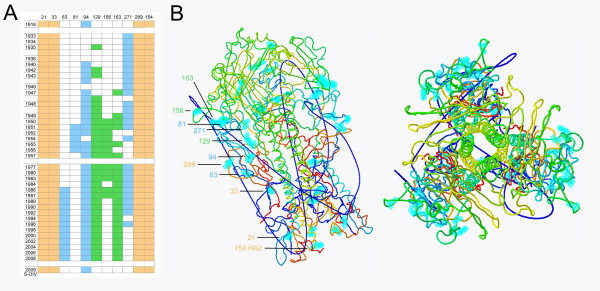
**Glycosylation of H1N1 viruses, 1918-2009**. A) HA glycosylation status of selected, relevant H1N1 viruses from 1918 to 2009 is shown. Beige coloration shows potential glycosylation sites in the fusion domain at positions 21, 33, 289, and 154 (HA2). Blue coloration represents glycosylation status at positions 63, 81, 94, and 271 in the vestigial esterase domain. Green coloration indicates glycosylation status at positions 129 (or 131), 156, and 163 on the globular head near the receptor binding site. The site labeled 129 had the attachment asparagine at position 131 through 1984, but at position 129 from 1986 to the present; these overlapping sites are mutually exclusive so are represented together. B) Structural representations of the H1N1 HA trimer based on the crystal structure of the 1918 pandemic strain [27]. Sites for potential glycosylation have been superimposed on the structure in light blue and labeled to correspond to the chart in Figure 3A.

N-Linked glycosylation sites on the HA molecule were identified in a temporal ordering of human H1N1 viruses (Figure 3A). Four sites in the fusion domain are conserved in all human H1N1 viruses. The 1918 pandemic strain, the progenitor of modern H1N1 viruses, had a single potential glycosylation site in the vestigial esterase domain of the HA. Several new sites for glycosylation appeared in the 1930s and 1940s during evolution and adaptation, but the general pattern before 1949 was that only one to three sites were variably present in any one strain, with most of them distant from the antigenic regions on the globular head of the HA (Figure 3B). Beginning in 1949, glycosylation sites near the receptor binding site were generally present, resulting in a total of eight to ten sites in the complete HA molecule. After re-emergence of H1N1 strains in 1977, a high number of sites were always present and little variation was seen among strains. Similar to the phylogenetic analysis, the HA of novel H1N1 most resembles the 1918 pandemic strain and strains circulating in the 1930s, since novel H1N1 HA contains only a single potential site of glycosylation in the vestigial esterase (Figure 3A). If glycoconjugates are present at many or all of the potential sites for glycosylation of the strains post 1948, and these shield the relevant protein epitopes, persons exposed to these strains are likely to be unable to generate antibodies that can recognize novel H1N1.

## Discussion

We propose that both the age distribution of novel H1N1 cases thus far and the unusual pattern of pathogenesis observed may be explained by the evolutionary accumulation of glycosylation sites on HA1 in human H1N1 viruses and the immune response to first exposure to these viruses, the so-called doctrine of Original Antigenic Sin (OAS) [15,16].

Influenza H1N1 viruses have co-circulated with H3N2 and B viruses since 1977. It is unlikely, therefore, that any H1N1 viruses now emerging into circulation would be immunologically similar to those currently circulating. Indeed, a recent study [17] reported that antibodies to novel H1N1 were entirely absent in the sera of children, but present in 6-9% of a sample of adults ages 18-64 (sic) and 33% of sera from persons > 60 years of age.

### Supporting evidence for the Integrated Position - by age group

#### The age group ≥ 61 years - The virtues of Antigenic Sin with the right antigens

The doctrine of Original Antigenic Sin states that an individual's first infection with an influenza A-type virus produces an indelible mark upon that person's immune system including an antibody response that persists for many years, possibly a lifetime. Though necessarily not a part of the original surmise, it is now believed that the OAS immune response includes a cytotoxic T-cell component [18]. Based on this doctrine, persons who first encountered influenza viruses in the 1930s and early 1940s should produce immune responses upon influenza virus infection cross-reactive to similar viruses, among which is novel H1N1 (Figure 2). Persons born after H1N1 viruses left circulation in 1957, including the period during which re-emergent H1N1 circulated, i.e., from 1977 onward [19,20], would not be expected to produce an OAS response that would include antibody cross-reactive to novel H1N1. Indeed, the presence of antibodies to novel H1N1 [17] and the fact that there are few clinical cases among those over 60 years of age suggests that a B-cell mediated antibody response to viruses that circulated prior to 1948 is probably present in those age ≥ 61. Figure 3 highlights (in green) three sites for potential glycosylation all of which lie within the so-called Sa antigenic region on the globular head of H1 [21]. The B-cell epitopes defined on the globular head of early H1N1 viruses including the 1918 strain [22] present a much closer match by amino acid sequence to 2009 novel H1N1 than do recent circulating seasonal H1N1 viruses suggesting the mechanism of this immunity (data not shown). Interestingly, a similar response was also observed in elderly persons over age 77 in the US and Canada relative to H3N2 viruses and the pandemic of 1968/9 [23]. We intend that one possible contribution of this paper be the suggestion that loss of antigenicity may occur by the addition of sites of glycosylation adjacent to structurally antigenic sequences in addition to the obvious restructuring of antigenic sites by direct amino acid substitution.

#### The age group < 32 years: Antigenic Sin with inappropriate antigens

The H1N1 virus that re-emerged into circulation in 1977 was most similar to those that circulated between 1948 and 1950. The year prior, 1947, was notable for a generalized failure of the seasonal flu vaccine [24], and it was concluded that a very large genomic change, ascribed to intrasubtypic reassortment, had taken place between 1943 and 1947. This change involved at least five antigenic sites. Mortality associated with pneumonia and influenza declined by 2/3 between 1937 and 1947. It did not decline further between 1947 and 1957, the pandemic year in which H2N2 viruses displaced H1N1 viruses.

The progressive N-linked glycosylation of sites near those antigenic to the host is an adaptive evolutionary strategy employed by influenza viruses [25]. The added carbohydrate molecules shield immunodominant sites permitting the adapting virus to escape preformed immunity in hosts previously exposed to less glycosylated surface proteins. As a counter-balance to this method of immune escape, however, highly glycosylated viruses are better recognized by innate defenses such as collectins, and are less virulent in the lower respiratory tract. The sequential addition of glycoconjugates to the virus during adaptation may therefore also contribute to the attenuation of later strains compared to their pandemic forebears [26].

The 1918 pandemic virus had a hemagglutinin (HA) protein that contained five sites at which post-translational modification by glycosylation could occur [27]. Of these sites for potential glycosylation, four were located in the fusion domain and highly conserved. The remaining one was in the vestigial esterase domain and appeared variably in viruses until about 1950 at which point it became evolutionarily fixed (see Figure 3A). Between 1918 and 1947, three new glycosylation sites appeared in circulating strains, all in areas where attachment of glycoconjugates might interfere with antibody binding. However, in most sequences of strains which circulated prior to 1949, only one to three sites were present in any single strain on the globular head. Beginning with the 1949-1950 viruses, the immediate ancestors of the H1N1 strains that re-emerged in 1977, eight to ten total potential glycosylation sites were present in these and all subsequent viruses, with four to six of these located on or near the globular head where they could interfere with antibody binding and immunity, including three within the Sa antigenic region. H3N2 viruses have gone through a similar progression of added glycosylation sites since their introduction in 1968 [25].

Although glycosylation of HA can modify antibody based immunity, CD8+ T-cell epitopes have been highly conserved since the 1930s [28,29], and therefore, T-cell based immunity should be unaffected by evolutionary changes in HA. Indeed, a recent analysis of both B-cell and T-cell epitopes of novel H1N1 demonstrates that only 31% of potential B-cell epitopes from novel H1N1 are conserved in recent seasonal strains (and these may be inaccessible due to shielding by glycoconjugates as we discuss here), while the majority (69%) of CD8+ T-cell epitopes are conserved [30]. Unpublished data from author JAM [Wanzeck K and McCullers JA, in preparation] demonstrate that the glycosylation status of the influenza virus hemagglutinin profoundly affects disease pathogenesis during sequential infection of mice. First infection with a highly glycosylated influenza virus leads to a sub-optimal antibody response that does not protect mice from re-infection with a poorly glycosylated variant of the same strain. Re-infected mice clear the challenge viruses but show severe morbidity and pathology following viral clearance attributable to aberrant CD8+ responses.

We propose that the relative paucity of HA glycosylation has two effects on the pathogenesis of novel H1N1. First, it has been demonstrated previously that lack of glycosylation contributes to virulence in the lung by precluding clearance by innate factors [25]. This likely contributed to the extreme virulence of the 1918 pandemic strain, wherein a large part of the virulence in animal models has been attributed to the HA and NA [31]. Similarly, novel H1N1 is highly virulent in animal models, a property linked to its ability to replicate efficiently in the lungs [32], and likely attributable to lack of glycosylation. Second, we suggest that Original Antigenic exposure to H1N1 viruses with highly glycosylated HA1 could generate an immune response similar to that seen in mice exposed to highly glycosylated mutants. This immune response would not protect from infection, but would permit viral clearing and introduce the possibility of CD8+ cell-mediated toxicity in some subset of those affected. Therefore, persons whose first exposure to H1N1 viruses was to the highly glycosylated re-emergent viruses that appeared in circulation after 1977 would have a much lower level of immunoprotection, and would be at risk for aberrant T-cell responses and severe lung disease from cytokine storm. In support of this thesis, an autopsy series of 21 patients with fatal novel H1N1 infection demonstrated severe lung pathology highlighted by an aberrant pulmonary immune response characterized by a massive infiltration of CD8+ T-cells into the lungs [33].

For this type of immuno-failure, a mismatch of antibody and T-cell response is required. Therefore, a gradation of responses would be expected within this age range. An individual exposed previously only to H1N1 viruses with highly glycosylated HA1 could have experienced the appropriate T-cell epitopes enough times to have a robust CD8+ response, but have little to no cross-protective antibody to novel H1N1. If such persons were exposed relatively infrequently and to few distinct variants of H1N1, the mismatch would be especially great. In our composite age distribution of cases, the peak occurs in the age range 10-19 years. A shift in case and age distributions to younger age appears to be a general characteristic of influenza pandemics [34], and there is now a general consensus that school age children may be important early spreaders of pandemic disease. However, there is more detailed work [e.g., [35]] that suggests that pre-school children should lead and possibly dominate case distributions. Therefore, while it may be that this extraordinary concentration derives primarily from the high social contact rate in this age group coupled with some proclivity of this particular influenza virus, we should also admit the possibility that additional immunological factors could be contributory. In the US between 1977 and 1988, H1N1 and influenza B viruses dominated in circulation in 8 of the 12 seasons. Between 1989 and 1998, H1N1 and B viruses dominated in only 3 of 10 seasons whereas from 1999 to 2008, H1N1 and B viruses dominated in 4 of 10 seasons and H1N1 viruses circulated widely but did not dominate in 2007/8 [36]. There is also evidence that genetic divergence within H1N1 viruses was lower in the 1990s than in either the 1980s or the first decade of the 21^st ^century [37]. Taken together, persons currently age 10-19 would have the smallest opportunity to encounter H1N1 viruses as their Original Antigenic Sin, and their exposure would have been to very similar viruses. Older individuals would have a greater opportunity for OAS exposure to more genetically diversified H1N1 viruses, though exposure for all of these would have been to a highly glycosylated HA. Young children, relatively naïve with respect to influenza, would have little opportunity to develop a robust T-cell response to H1N1, and no prospect for antibodies neutralizing to novel H1N1. They could be productively infected producing mild disease without the possibility of T-cell mediated immunopathology. A recent study, also in mice [38], demonstrated that regulation of the cytotoxic T-cell response in highly pathogenic influenza infection may require a fine hand for effective modulation. Complete suppression of the cells required for proliferation of CD8+ T-cells in the infected lung was not effective in reducing morbidity or mortality; but modulating the facilitating cell population using a widely available and inexpensive human pharmaceutical did substantially mitigate the impact of infection.

In summary, persons aged ≤ 32 years are expected to have an OAS experience restricted to highly glycosylated H1N1 viruses and would thereby be expected to have inadequate immuno-protection for exposure to novel H1N1. Furthermore, the subgroup of age 10-19 years could have had an especially low rate of OAS exposure to any H1N1 virus. If the mouse data map onto humans, an OAS response conditioned by a highly glycosylated H1N1 virus could be responsible for the morbidity seen in a subgroup of younger persons. About one-half of cases with severe pneumonia in Mexico had no underlying medical condition; and in a detailed study of a subset of these cases, no evidence was found for bacterial pneumonia as a co-morbid condition [39,40]. The evidence we cite suggests that such cases should be examined for cytotoxic T-cell activity; and considered for experimental therapy with likely modifiers of the influenza-specific inflammatory process, such as agonists of peroxisome proliferator-activated receptor-γ [38,41]

#### The age group, 32< age < 61 years: Experience, with a tincture of time

In our composite of countries, the incidence of cases in these age groups declined curvilinearly to very low levels. Persons in a subgroup, aged 52-61 years, were born during the last decade of H1N1 dominance. The H1N1 viruses of 1947 to 1957 were more highly glycosylated than those of earlier years, but there was considerable heterogeneity and genetic divergence making this exposure somewhat rich. In particular, the sites for potential glycosylation varied more significantly during this period than after the re-emergence in 1977 (Figure 3A). It seems reasonable, therefore, that immunoprotection derived from OAS exposure in this subgroup would be considerable, if distinctly lower than in those over age 61. The associated experience is captured in the age group 50-59 (Figure 1).

It is more difficult to argue that those aged 32 to 52 years should have an acquired immunoprotection different from those age <32 years, since none born between 1957 and 1977 would have had exposure to an H1N1 virus as an OAS experience. We note, however, that three of the eight genes in novel H1N1 (M, NP, NS) are cited as derived from the common H1N1 ancestral line and a fourth, PB1, is thought to have been reassorted from human H3N2 viruses and thence from an avian precursor [42]. Persons aged 32-51, therefore, would have had greater cumulative immunological experience with variation in 4 of the gene segments currently found in novel H1N1. It has also been previously noted that infection with H3N2 viruses is somewhat protective against reinfection with H1N1 viruses and vice versa [43] This less specific immunological experience coupled with a non-H1N1 OAS response appears to have produced a susceptibility to novel H1N1 intermediate between the two age groups with a direct OAS response to H1N1 viruses.

## Conclusions

We have suggested here that the doctrine of Original Antigenic Sin [44] modified by glycosylation may explain the age-specific patterns of clinical attack and toxicity seen in the now waning novel H1N1 pandemic season. We have invoked the colorful historical label, OAS, directly, to characterize the apparent relative low level of novel H1N1 infection in persons over age ~ 60 years noted now by a wide variety of observers. We have also sought to extend the doctrine and to make it more specific. The first extension is to relate evolutionary changes in the viral sequences for HA to specific age ranges. The second is to broaden the concept of antigenic drift to include the arising of sites of potential glycosylation in and near regions known to be responsible for neutralizing antigenicity. The first proposed extension might be validated by data collection and epidemiological modeling of age-specific susceptibility and case fatality ratios. The second will require studies of escape mutants generated in a human-specific glycosylation environment. A further extension is our suggestion that OAS responses to viruses of similar type but evolutionarily and antigenically distinct may be responsible for the unusual toxicity seen in young persons with novel H1N1 infection. Validation of this suggestion would entail the isolation and characterization of infiltrating lymphocytes and dendritic cells in persons with such infections; and might proceed empirically by careful and controlled clinical investigation in critically ill patients of agents (widely available and non-toxic) shown to modify levels of such infiltrating cells in animal models. The low level of virulence of the novel H1N1 pandemic has provided us with a splendid opportunity to exercise the machinery of modern science to investigate the characteristics of pandemic viruses without the usually attendant elevated levels of mortality and morbidity.

### The importance of the perspective presented here

1. The principle of Original Antigenic Sin modified by the immunological response to glycosylation of antigenic sites throughout the evolution of circulating influenza viruses provides a consistent and arguably complete explanation for the age distribution of cases observed in the novel H1N1 pandemic to date.

2. This integration points to a specific class of immunological phenomena as the likely cause of severe disease in the absence of underlying conditions for the immediate focus of clinical and laboratory investigation.

3. If the investigation of cases of severe morbidity should permit the identification of a specific cellular agent as the likely cause, specific therapeutic actions can be enjoined ranging from the inhibition of a specific modulator of influenza infection to the removal or inactivation of specific cell types.

4. It informs administrative strategy with regard to the distribution of scarce pharmaceutical resources (vaccines and antivirals) to younger age groups and the selection of and emphasis upon the use of non-pharmaceutical measures most appropriate to the affected age groups, e.g., school closures, discouraging alternative social gatherings, augmented and continuing mask wearing in the young, etc.

5. The ongoing pandemic is an extraordinary opportunity to validate, and possibly extend the doctrine of Original Antigenic Sin

## List of Abbreviations

**Novel H1N1**: The A(H1N1) influenza virus of swine-origin that has emerged in the pandemic of 2009; **H1N1**: A(H1N1) viruses other than novel H1N1; **H2N2**: A(H2N2) influenza viruses; **H3N2**: A(H3N2) influenza viruses; **HA**: the hemagglutinin protein of influenza A viruses; **HA1**: the globular head of the hemagglutinin protein; also know as subunit 1; **N-X-S/T**: The amino acid subsequence, asparagine-X-serine or threonine, where X is Not proline; **OAS**: Original Antigenic Sin.

## Competing interests

The authors stipulate that they know of no potential competing interests to the material and presentation in this manuscript.

## Authors' contributions

TR, JM and RC conceived the study. JM prepared and analyzed the phylogenetic tree and glycosylation site chart of influenza H1N1 viruses. GC and HN compiled and investigated the age-specific epidemiological data. TR drafted the manuscript, and JM, GC, HN and RC subsequently commented on it. All authors read and approved the final manuscript.

## Pre-publication history

The pre-publication history for this paper can be accessed here:

http://www.biomedcentral.com/1471-2334/10/5/prepub

## Supplementary Material

Additional file 1 **Data underlying **Figure 1. The data used to construct Figure is tabulated. In addition, the file includes data from the United States Click here for file

Additional file 2 **Supplementary Data on HA sequences**. Each HA sequence is labeled, together with the year of origin and the accession number Click here for file
