# Supplementary material for: Does Glycosylation as a modifier of Original Antigenic Sin explain the case age distribution and unusual toxicity in pandemic novel H1N1 influenza?
Source: BMC Infect Dis. 2010 Jan 7;10:5. doi: 10.1186/1471-2334-10-5 (PMC3003248; doi:10.1186/1471-2334-10-5)
Supplement: Additional file 2 — Supplementary Data on HA sequences. Each HA sequence is labeled, together with the year of origin and the accession number [file 1471-2334-10-5-S2.DOC]

**Table. Virus strains utilized in this report.**

______________________________________________________________________

**Year Name HA Accession Number**

______________________________________________________________________

1918 A/Brevig Mission/1/1918 AF116575

1933 A/Wilson Smith/1933 DQ508905

1934 A/Puerto Rico/8/1934 CY009444

1935a A/Alaska/1935 CY019955

1935b A/Philadelphia/1935 CY020469

1936 A/Henry/1936 CY020445

1940 A/Hickox/1940 CY013271

1942 A/Bel/1942 CY009276

1943a A/Weiss/1943 AF494247

1943b A/Marton/1943 AF494248

1946 A/Cam/1946 CY009596

1947a A/Rhodes/1947 AF494249

1947b A/Fort Monmouth/1/1947 AF494250

1948a A/Lepine/1948 AB043479

1948b A/Albany/4835/1948 CY019947

1949 A/Roma/1949 CY019971

1950 A/Albany/4836/1950 CY021701

1951 A/Tokyo/1/1951 AB043481

1952 A/Kojiya/1/1952 AB043482

1954a A/Taiwan/13/1954 AB043483

1954b A/Malaya/302/1954 CY021053

1955 A/Yamagishi/1955 AB043484

1956 A/Meguro/1/1956 AB043485

1957 A/Saga/2/1957 AB043486

1977 A/Hong Kong/117/77 CY009292

1978 A/Memphis/15/1978 CY010892

1979 A/Memphis/1/1979 CY019739

1980 A/Memphis/7/1980 CY010908

1981 A/Baylor/4052/1981 CY021029

1982 A/Baylor/11735/1982 CY009620

1983 A/Memphis/12/1983 CY010948

1984 A/Finland/5/1984 L33492

1986 A/Memphis/12/1986 CY019101

1987 A/Memphis/4/1987 CY019779

1990 A/Texas/22/1990 L19020

1991 A/Texas/36/1991 DQ508889

1992 A/Aichi/24/1992 AB043490

1994 A/Hong Kong/59/1994 AJ457901

1995 A/New York/615/1995 CY010508

1996 A/New York/646/1996 CY013287

1998 A/Hong Kong/1035/1998 AF386777

1999 A/New Caledonia/20/1999 CY033622

2000 A/Canterbury/100/2000 CY010132

2001 A/New York/342/2001 CY003320

2002 A/New York/291/2002 CY003304

2003 A/New York/348/2003 CY002704

2005 A/Denmark/33/2005 EU097949

2006 A/Mississippi/02/2006 EU199341

2008 A/Colorado/01/2008 EU716526

2009 A/California/4/2009 GQ117044

_____________________________________________________________________
