# Supplementary material for: Does Glycosylation as a modifier of Original Antigenic Sin explain the case age distribution and unusual toxicity in pandemic novel H1N1 influenza?
Source: BMC Infect Dis. 2010 Jan 7;10:5. doi: 10.1186/1471-2334-10-5 (PMC3003248; doi:10.1186/1471-2334-10-5)

	North America							EU		South America						Oceania				Asia							% under30	over65	
	USA			Canada		Mexico		EU		Argentina		Chile		Peru		Australia		New Zealand		Japan		Thailand		Composite			72.6330003	1.38536322	1
Age	Cases	Frequency	Age	Cases	Frequency	Cases	Frequency	Cases	Frequency	Cases	Frequency	Cases	Frequency	Cases	Frequency	Cases	Frequency	Cases	Frequency	Cases	Frequency	Cases	Frequency	Cases	Frequency		73.1123162	2.80306086	2
<5	4816	13.0056711	< 10	840	22.1434316	4480	28.1336348	1458	22.2256098	622	28.4798535	3852	33.8339921	549	31.0696095	402	21.6129032		21.5408805	909	19.3857965	1652	23.5193622	14901	26.6935187		78.5104245	1.53855815	3
5-24	22080	59.6273292	10-19	1406	37.0657551	5182	32.5420749	2123	32.3628049	523	23.9468864	3000	26.3504611	616	34.8613469	792	42.5806452		29.8742138	2191	46.7263809	3351	47.7078588	19374	34.7066159		77.6067073	1.98170732	4
25-49	7434	20.0756144	20-29	527	13.9031295	2840	17.8347149	1510	23.0182927	283	12.9578755	1306	11.4712341	247	13.9784946	299	16.0752688		22.6415094	816	17.4024312	950	13.5250569	8922	15.9835287	77.3836632	65.3846154	5.72344322	5
50-64	2187	5.90602214	30-39	304	8.0224639	1523	9.56417985	605	9.22256098	266	12.1794872	1043	9.16117699	165	9.33786078	152	8.17204301		10.3773585	365	7.78417573	456	6.49202733	4945	8.85902314		71.6556873	5.58629776	6
>=65	513	1.38536322	40-49	395	10.4179934	1071	6.72569706	470	7.16463415	206	9.43223443	857	7.5274484	101	5.71590266	116	6.23655914		8.80503145	226	4.819791	324	4.61275626	3822	6.84710207		79.909451	1.3016412	7
			50-59	214	5.64416561	583	3.66114042	264	4.02439024	159	7.28021978	691	6.06938955	66	3.73514431	72	3.87096774		5.18867925	132	2.81509917	189	2.69077449	2403	4.30492684		80.2688172	1.4516129	8
			>=60	106	2.80306086	245	1.53855815	130	1.98170732	125	5.72344322	636	5.58629776	23	1.3016412	27	1.4516129		1.57232704	50	1.06632544	102	1.45216401	1454	2.6052847		74.0566038	1.57232704	9
	37030	100		3794	100	15924	100	6560	100	2184	100	11385	100	1767	100	1860	100		100	4689	100	7024	100	55823			83.5146087	1.06632544	10
																											84.7522779	1.45216401	11
US data appears courtesy Fiore A. Presentation at ACIP, 29 July 2009																		New Zealand has asked that we display only the frequency and not the case numbers											
																											76.4913191	2.35113647	

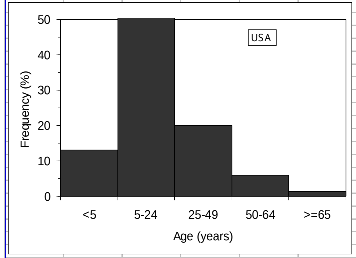

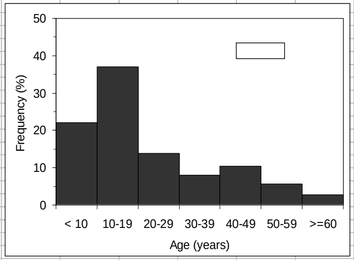

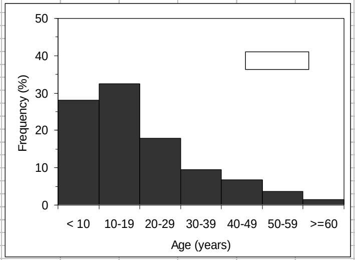

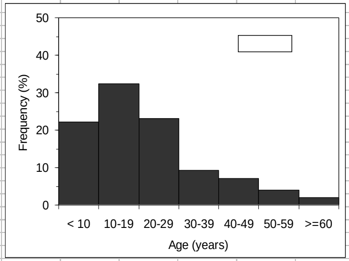

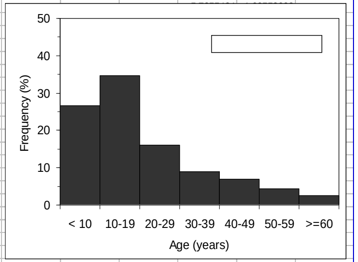

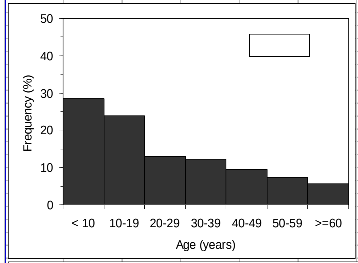

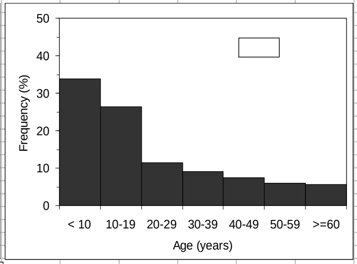

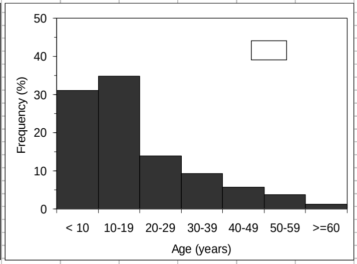

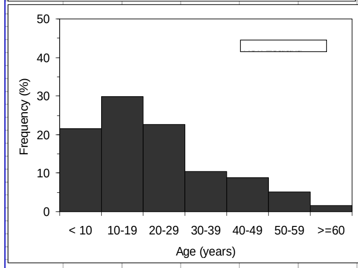

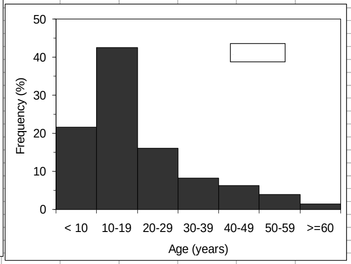

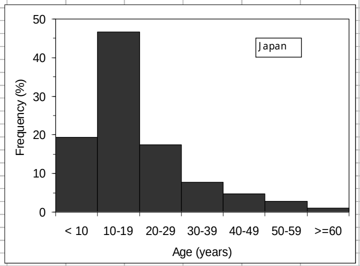

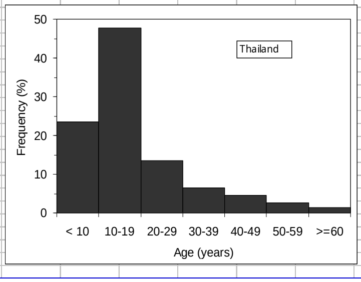

Supplement: Supplementary file 2 [file 1471-2334-10-5-S1.DOC]
